# A Practical Treatise on Materia Medica and Therapeutics

**Published:** 1900-07

**Authors:** 


### Books and Magazines.

A PRACTICAL TREATISE ON MATERIA MEDICA AND THERAPEUTICS. —By Roberts Bartholow, M. A., M. D., LL. D., Professor Emeritus of Materia Medica, General Therapeutics and Hygiene in the Jefferson Medical College of Philadelphia, etc., etc., etc. Tenth edition, revised and enlarged. Price, in cloth, $5.00; sheep, $6.00. D. Appleton & Co., publishers, New York.

Of the many excellent works on materia medica and therapeutics none have enjoyed a greater popularity with American physicians and medical students than this one. The first edition, published in 1876, was received with unusual favor, and, because of its special arrangement and its peculiar adaptability to the wants of medical students, his work has continued to hold a place in the front rank in substantial appreciation by the profession. Bartholow's Materia Medica and Therapeutics is so well and favorably known to physicians that it seems almost unnecessary to again call attention to it except to make known the fact that it is being kept abreast of the progress in this branch of medicine by frequent revisions, and to indicate some of the more important changes and additions that are being made.

In this edition the same scheme of classification has been followed as in former editions, part first treating of the routes by which medicines are introduced into the organism; part second, the action and uses of remedial agents— those used to promote constructive metamorphosis; those promoting destructive metamorphosis or increasing waste; remedies used to destroy microbes, or morbific germs, and to prevent or arrest septic processes; agents used to modify the functions of organs; remedies which diminish or suspend the functions of the cerebrum after a preliminary stage of excitement; agents which depress the motor functions of the spinal cord and sympathetic; and part third treating of topical remedies.

The principal changes made in this edition are the addition of an article on prescription writing, accounts more or less full of the newer remedies, and striking out of references, thus saving considerable space.

This edition will no doubt meet with the same favorable reception that has been accorded to its predecessors, and which it so abundantly merits. S. E. H.

